# Functional and structural analysis of a novel splice site *HMBS* variant in a Chinese AIP patient

**DOI:** 10.3389/fgene.2023.1333111

**Published:** 2023-12-19

**Authors:** Xiaoqing Wang, Huifen Zhang, Huanhuan Huang, Wenli Wang, Yuping Wen, Zhuojin Dai, Shuling Huang, Jingyi Zhou, Yuqing Zhou

**Affiliations:** ^1^ Dongguan Hospital, Guangzhou University of Chinese Medicine, Dongguan, Guangdong Province, China; ^2^ Department of Endocrinology, Dongguan Hospital of Guangzhou University of Chinese Medicine, Dongguan, Guangdong Province, China; ^3^ The First Clinical Medical College, Guangdong Medical University, Zhanjiang, Guangdong Province, China

**Keywords:** acute intermittent porphyria, HMBS, novel splice mutation, non-sense-mediated mRNA decay, pathogenicity of the mutation, molecular mechanism of the mutation

## Abstract

**Background:** Acute intermittent porphyria (AIP) is a rare metabolic disorder that results from mutations in the gene encoding hydroxymethylbilane synthase (HMBS), an enzyme involved in heme biosynthesis. AIP follows an autosomal dominant inheritance pattern, but most carriers are asymptomatic. The clinical manifestations of AIP include acute attacks of abdominal pain and neuropsychiatric disturbances. The pathogenicity of novel *HMBS* variants identified in Chinese patients has not been well established.

**Objective:** The article aims to identify the pathogenic mutation in an AIP patient and prove its pathogenicity through *in vitro* experiments.

**Methods:** A 22-year-old female diagnosed with AIP participated in the study. Variant screening of her *HMBS* gene was carried out through Sanger sequencing. To ascertain the consequences of the newly discovered variant, we conducted *in vitro* experimentation targeting *HMBS* gene expression and enzymatic function. Additionally, protein structure analysis was performed. Cycloheximide treatment and *UPF1*-specific siRNA knockdown were employed to assess the impact of the mutation on the mechanism of non-sense-mediated mRNA decay (NMD).

**Results:** A novel splice site variant in the *HMBS* gene (c.648_651+1delCCAGG) was detected in the patient, which caused aberrant mRNA splicing. *In vitro* experiments demonstrated that this variant significantly decreased the expression of HMBS. Further investigation confirmed that this decrease was due to NMD. Additionally, structural analysis indicated that this variant would destabilize the HMBS protein and impair its catalytic activity. To gain a comprehensive understanding of *HMBS* mutations in the context of AIP, we conducted a literature search on PubMed using the keywords ‘*HMBS*’ and ‘Acute intermittent porphyria’ from 2013 to 2023. This search yielded 19 clinical case reports written in English, which collectively described 220 *HMBS* gene mutations worldwide.

**Conclusion:** The study identified and proved the pathogenicity of a novel splice site *HMBS* variant for the first time. Our results elucidated the pathological mechanism by which this mutation causes AIP through reducing HMBS expression and activity. These findings provide theoretical guidance for the diagnosis, treatment and genetic counseling of AIP patients.

## Introduction

Acute intermittent porphyria (AIP) is a rare inherited metabolic disorder caused by hydroxymethylbilane synthase (HMBS) deficiency, the third enzyme in the heme biosynthetic pathway. It belongs to porphyrias, a group of rare genetic metabolic disorders that can be classified into acute and/or cutaneous types based on their clinical manifestations (Karim et al., 2015). HMBS deficiency leads to the accumulation of porphyrin precursors and porphyrins in the body. AIP is autosomal dominant, but has low clinical penetrance. Most *HMBS* mutation carriers are asymptomatic (Bustad et al., 2021). An epidemiological survey of AIP in Hebei Province, China, reported a prevalence of pathogenic *HMBS* mutations of 1/1,765 and an annual incidence of 0.07 (95% CI: 0.03–0.17) to 0.08 (95% CI: 0.03–0.18) per million in 2018 and thereafter (Ma et al., 2022). The estimated penetrance in known AIP families was about 22.9% (Lenglet et al., 2018). AIP inheritance is modulated by environmental and/or other genetic factors in *HMBS* variant carriers, rather than following the classic autosomal dominant model (Lenglet et al., 2018). AIP symptoms usually develop after puberty and are mainly severe abdominal pain and neuropsychiatric symptoms (Whatley et al., 1993). AIP can also cause long-term complications such as hepatocellular carcinoma, hypertension, and renal failure (Andersson et al., 2000; Pallet et al., 2015; Baravelli et al., 2017). Acute attacks are more frequent in females. Precipitating factors include drugs that affect the heme biosynthetic pathway, steroids hormones, nutritional status changes, and infections that trigger a stress response (Ramanujam and Anderson, 2015; Stolzel et al., 2019). AIP diagnosis mainly relies on urine tests to detect porphyrin precursors (delta-aminolevulinic acid and porphobilinogen) and porphyrins levels. During acute attacks, these levels increase significantly, turning the urine from red to brownish red. Serum porphobilinogen levels, erythrocyte porphobilinogen deaminase activity, or gene sequencing can also be used to confirm the diagnosis (Anderson et al., 2005). Currently, the main therapeutic approach for AIP focuses on traditional treatments that aim to improve acute symptoms, with hemin being the most effective (Schmitt et al., 2018; Blaylock et al., 2020). In recent years, some therapies based on pathogenesis have shown promise in development (Fontanellas et al., 2019; Wang et al., 2019), especially givosiran, a N-acetylgalactosamine (GalNAc)-conjugated RNA interference (RNAi) drug, which has remarkable efficacy (Balwani et al., 2020).

A 22-year-old pregnant woman with an acute AIP attack was reported. Genetic testing identified a novel splicing mutation (c.648_651+1delCCAGG) in exon 10 of *HMBS* gene. The Minigene technique was employed in this study to investigate the impact of gene mutations on pre-mRNA alternative splicing. This was achieved by transfecting cells with plasmid vectors containing exon fragments harboring mutant genes, such as intron retention and exon skipping. Abnormal mRNA products were subsequently detected using QPCR and Sanger sequencing. Through this approach, the clinical relevance of certain pathogenic mutations was elucidated (Fraile-Bethencourt et al., 2019; Putscher et al., 2021). This mutation impaired mRNA splicing and resulted in a truncated protein (p.Gln217Thrfs*33) missing 144 amino acids. Its pathogenicity was confirmed by *in vitro* assays. This case contributed to the diagnosis and genetic counseling of AIP and the mutation spectrum of *HMBS* gene.

## Materials and methods

### Patient’s clinical phenotype

A 22-year-old pregnant woman with severe abdominal and back pain was reported. She had spontaneous symptoms without clear triggers a week before admission and developed epileptic seizures and lower limb weakness 3 days later. She had a normal pregnancy and delivery history, but reported mild abdominal and back pain around menstruation. She and her family had no other relevant medical history. She had hypertension and tachycardia on physical examination. Her abdomen was non-tender and her limbs had normal muscle tone. Her upper limb muscle strength was 5/5 and her lower limb muscle strength was 3/5. She had hyponatremia and liver dysfunction on laboratory tests. Urinalysis confirmed the diagnosis of AIP. We sequenced the *HMBS* (NM_000190.4) gene using the 3730XL DNA Analyzer (Applied Biosystems, Foster City, California) and identified a heterozygous mutation (c.648_651+1delCCAGG) in exon 10 of the *HMBS* gene (Zhou et al., 2022).

### *In Vitro* expression vector construction

The SalI-wt-NotI fragment was amplified using the full-length synthetic *HMBS* CDS as a template and PHAGE-HMBS-SalI-F (primer sequence: TGACGTCGACCATGTCTGGTAACGGCAATGC)/PHAGE-HMBS-NotI-R (primer sequence: CGA​CGC​GGC​CGC​TTA​ATG​GGC​ATC​GTT​AAG​CTG) as primers. It was inserted into the PHAGE vector after double digestion with SalI and NotI restriction enzymes, resulting in the wild-type PHAGE-HMBS-wt vector, which was verified by sequencing. The mut-1, mut-2 and mut-3 fragments were amplified using the primer pairs PHAGE-HMBS-SalI-F (primer sequence: TGACGTCGACCATGTCTGGTAACGGCAATGC)/HMBS-E9-E10-R (primer sequence: CCT​CAG​GGT​GCA​GGA​TCT​GCC​CCA​CCC​GGT​T), HMBS-E9-E10-F (primer sequence: AACCGGGTGGGGCAGATCCTGCACCCTGAGG)/HMBS-E11-E12-R (primer sequence: ACT​GCA​GCC​TCC​TTC​CAG​GTG​CCT​CAG​GAA) and HMBS-E11-E12-F (primer sequence: TTCCTGAGGCACCTGGAAGGAGGCTGCAGT)/PHAGE-HMBS-NotI-R (primer sequence: CGA​CGC​GGC​CGC​TTA​ATG​GGC​ATC​GTT​AAG​CTG), respectively. Purified PCR products were mixed at a ratio of 1:1:1 as a template and amplified with PHAGE-HMBS-SalI-F/PHAGE-HMBS-NotI-R as primers to obtain the SalI-mut-NotI fragment. It was inserted into the PHAGE vector after double digestion with SalI and NotI restriction enzymes, resulting in the mutant PHAGE-HMBS-mut vector, which was verified by sequencing.

### Cell transfection and protein expression in HEK293T cells

HEK293T cells were cultured in DMEM medium containing 10% fetal bovine serum. The same amount of wild-type and mutant eukaryotic recombinant expression vectors were transfected into HEK293T cells according to the instructions of Hieff Trans (YEASEN, Cat. No. 40802ES03). Samples were collected 48 h after transfection for Quantitative real-time PCR (QPCR) detection and Western blotting detection. Total RNA was extracted using the Trizol method (TaKaRa, Cat. No. 9109) and cDNA was synthesized after digesting the DNA. QPCR detected the expression levels of the wild-type and mutant target genes. Total protein was extracted from the cells using RIPA lysis buffer (Beyotime, Cat. No. P0013). The protein concentration was determined using the BCA Protein Quantification Kit (YEASEN, Cat. No. 20201ES76). Equal amounts of total protein were subjected to SDS-PAGE electrophoresis. Western blottiong was performed to detect the expression levels of wild-type and mutant target proteins. Equal amounts of protein from each sample and prestained marker were loaded for electrophoresis. The proteins were then transferred to PVDF membranes, blocked with blocking solution, and incubated with primary antibody (DIAAN, HA antibody, Cat. No. 3063) and secondary antibody (ABclonal, HRP Goat Anti-Mouse IgG (H + L), Cat. No. AS003). Finally, enhanced chemiluminescence was used for staining, and the chemiluminescence imaging system (Syngene Bio Imaging Systems) was used to detect the fluorescence signal.

### Cycloheximide treatment and expression analysis after transfection

The presence of NMD impedes the identification of aberrantly spliced mRNA and consequently hinders the recognition of variants that modify canonical splicing. However, the application of cycloheximide to inhibit mRNA degradation may enable the determination of alternatively spliced mRNA transcripts that would otherwise remain undetectable (Schneider-Poetsch et al., 2010). After transfecting HEK293T cells with the same amount of wild-type and mutant eukaryotic recombinant expression vectors PHAGE-HMBS-wt and PHAGE-HMBS-mut for 40 h, cycloheximide (CHX) with 500 mM (MedChemExpress, Cat. No. HY-12320) was added and treated for 8 h. Cell samples were collected and total RNA was routinely extracted using the Trizol method. QPCR is used for evaluating expression levels of the wild-type and mutant *HMBS* mRNA. Primers phage-HMBS-qpcr-F (5′-AGA​TTA​CGC​TGG​ATC​CGC​TA-3′) and phage-HMBS-qpcr-R (5′-TCA​ATG​TTG​CCA​CCA​CAC​TG-3′) were used to detect the expression levels of *HMBS* in wt and mut samples.

### *UPF1*-specific siRNA and expression analysis

We used *UPF1* siRNA to diretly inhibit NMD (Park et al., 2020). The same amount of wild-type and mutant eukaryotic recombinant expression vectors PHAGE-HMBS-wt and PHAGE-HMBS-mut were co-transfected into HEK293T cells with *UPF1* small interfering RNA (interference target sequence: GAC​TCT​GGT​AAT​GAG​GAT​TTA) (50 nmol/mL), respectively. Cell samples were harvested 48 h later for QPCR and WB detection. Total RNA was routinely extracted using the Trizol method. QPCR is used for evaluating expression levels of the wild-type and mutant *HMBS* mRNA. Primers phage-HMBS-qpcr-F (5′-AGA​TTA​CGC​TGG​ATC​CGC​TA-3′) and phage-HMBS-qpcr-R (5′-TCA​ATG​TTG​CCA​CCA​CAC​TG-3′) were used to detect the expression levels of HMBS in wt and mut samples. Primers UPF1-QPCR-F (5′-AGA​GGT​GAC​CCT​GCA​CAA​GG-3′) and UPF1-QPCR-R (5′-AGC​CGA​GGA​GGA​AGA​CGT​TG-3′) were used to detect *UPF1* mRNA in wt and mut samples treated with siRNA *UPF1*. Total protein was routinely extracted from the cells using RIPA lysis buffer. The protein concentration was determined using the BCA Protein Quantification Kit. Equal amounts of total protein were subjected to SDS-PAGE electrophoresis. WB detected the expression levels of wild-type and mutant proteins.

### Prokaryotic expression and purification of the target protein

We used *Escherichia coli* T7 Express strain as the host and induced the expression of the target protein with IPTG (1 mM). The induction conditions were: cell density OD600 = 0.6, temperature 37°C, time 4 h. Then, we balanced and washed the Ni2+ resin with PBS (pH = 7.4) and eluted the target protein with different concentrations of imidazole (30 mM, 50 mM, 300 mM).

### Enzyme activity assay

The same amount of wild-type and mutant eukaryotic recombinant expression vectors PHAGE-HMBS-wt and PHAGE-HMBS-mut were transfected into HEK293T cells. Cells were collected 48 h later for enzyme activity assay according to the instructions of the human hydroxymethylbilane synthase enzyme activity assay kit (JINGMEI BIOTECHNOLOGY, Cat. No. JM-5895H2). The assay involved dilution of standards, sample loading, incubation, washing, enzyme addition, color development, termination and measurement. After thorough washing, the antibody-antigen complex was added with 3,3′,5,5′-Tetramethylbenzidine (TMB) substrate for color development. TMB was converted to blue and then to yellow under acid by horseradish peroxidase (HRP) catalysis. The color intensity was directly proportional to the hydroxymethylbilane synthase in the sample. The optical density (OD value) was measured at 450 nm wavelength by an enzyme-linked analyzer. The activity concentration of hydroxymethylbilane synthase in the sample was calculated from the standard curve.

### Protein structure prediction and thermal stability analysis

The structures of wild-type and mutant proteins were predicted using the I-TASSER online prediction website (I-TASSER: https://seq2fun.dcmb.med.umich.edu//I-TASSER/ https://zhanggroup.org/I-TASSER/). Protein structures were visualized by Pymol software (The PyMOL Molecular Graphics System, Version 1.8.4.0.) with default parameters. Thermal denaturation data of differential scanning fluorimetry (DSF) were obtained by a differential scanning fluorimeter (Prometheus NT.48, Nanotemper). Protein Mut and Wt samples had concentrations of about 0.782 mg/mL and 0.907 mg/mL, respectively, and were in 1X PBS buffer. All samples had a temperature range of 20°C–90 °C and a heating rate of 1 °C/min. Each protein was repeated three times per group.

## Result

### Construction of expression vectors and analysis of protein expression

**FIGURE 1:**
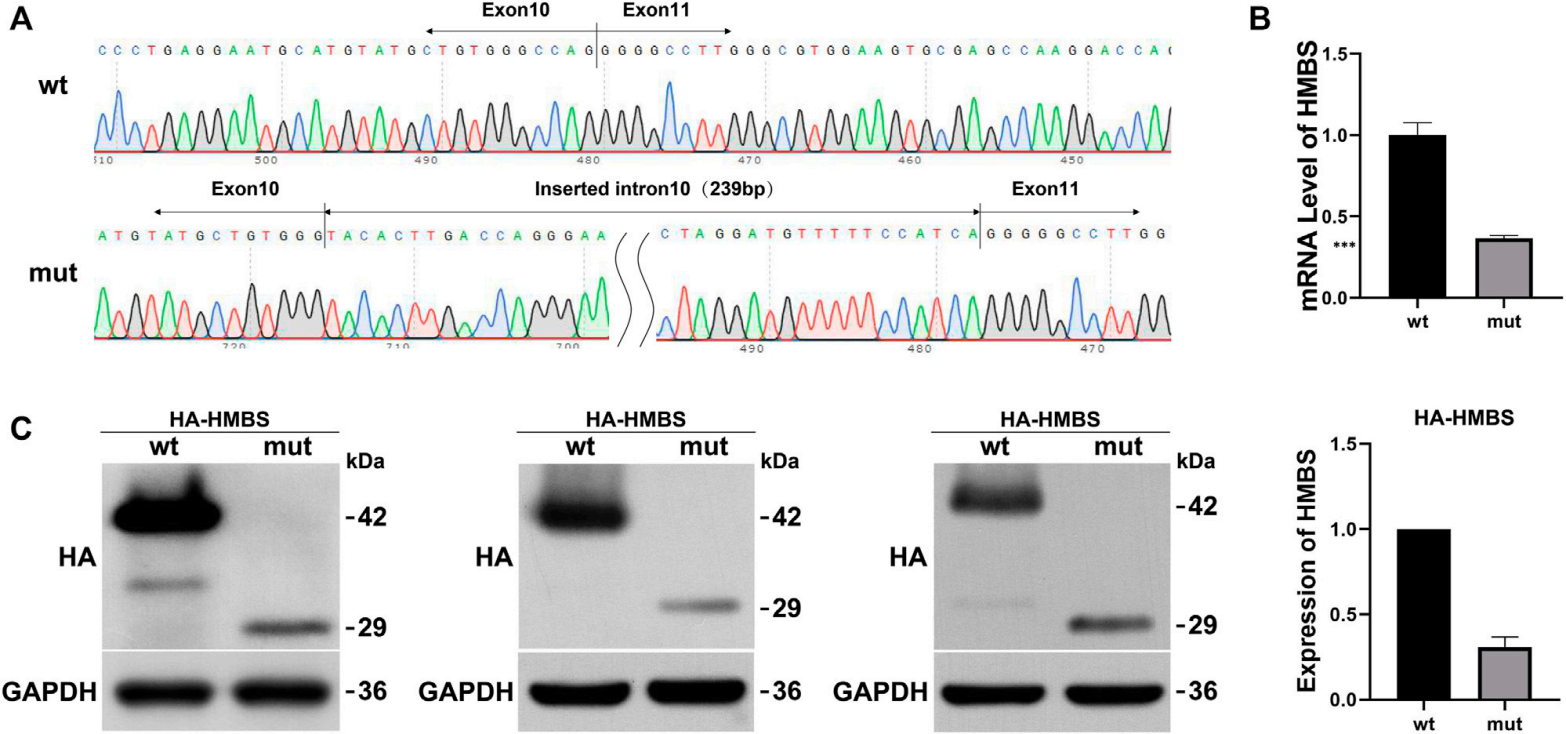
The effect of mutation on HMBS expression. **(A)** HMBS-wt/mut sequencing results show the successful construction of vectors. The mutated transcript revealed an insertion of 239 bp between exons 10 and 11; **(B)** Relative mRNA levels of *HMBS* mutant and wild type; **(C)** Western blot staining of wild-type and splice site mutant highlight relative protein amount. Additionally, the bar graph provides a visual representation of the relative expression levels of wild-type and mutant HMBS protein.

Wild-type and mutant eukaryotic HMBS expression vectors were constructed (Figure 1A) and transfected into HEK293T cells. mRNA and protein expression levels were measured and the results were as follows: expression levels of wild-type and mutant transcripts were detected by primers HMBS-PHAGE-qpcr-F and HMBS-PHAGE-qpcr-R. The mut expression was 0.37 compared with the wt control (Figure 1B). HA tag antibody was used to measure expression levels of wt and mut proteins. The wild-type protein had a size of 42 kDa, while the mutant mut had a size of 29 kDa. WB results showed that the mutant had a lower protein expression level than the wild type (Figure 1C). The expression levels of mRNA and protein in the mutant were significantly downregulated after *in vitro* expression detection, suggesting that the mutation generated a premature termination codon PTC, which might undergo NMD degradation.

### Analysis of results after cycloheximide treatment and siRNA *UPF1* treatment

**FIGURE 2:**
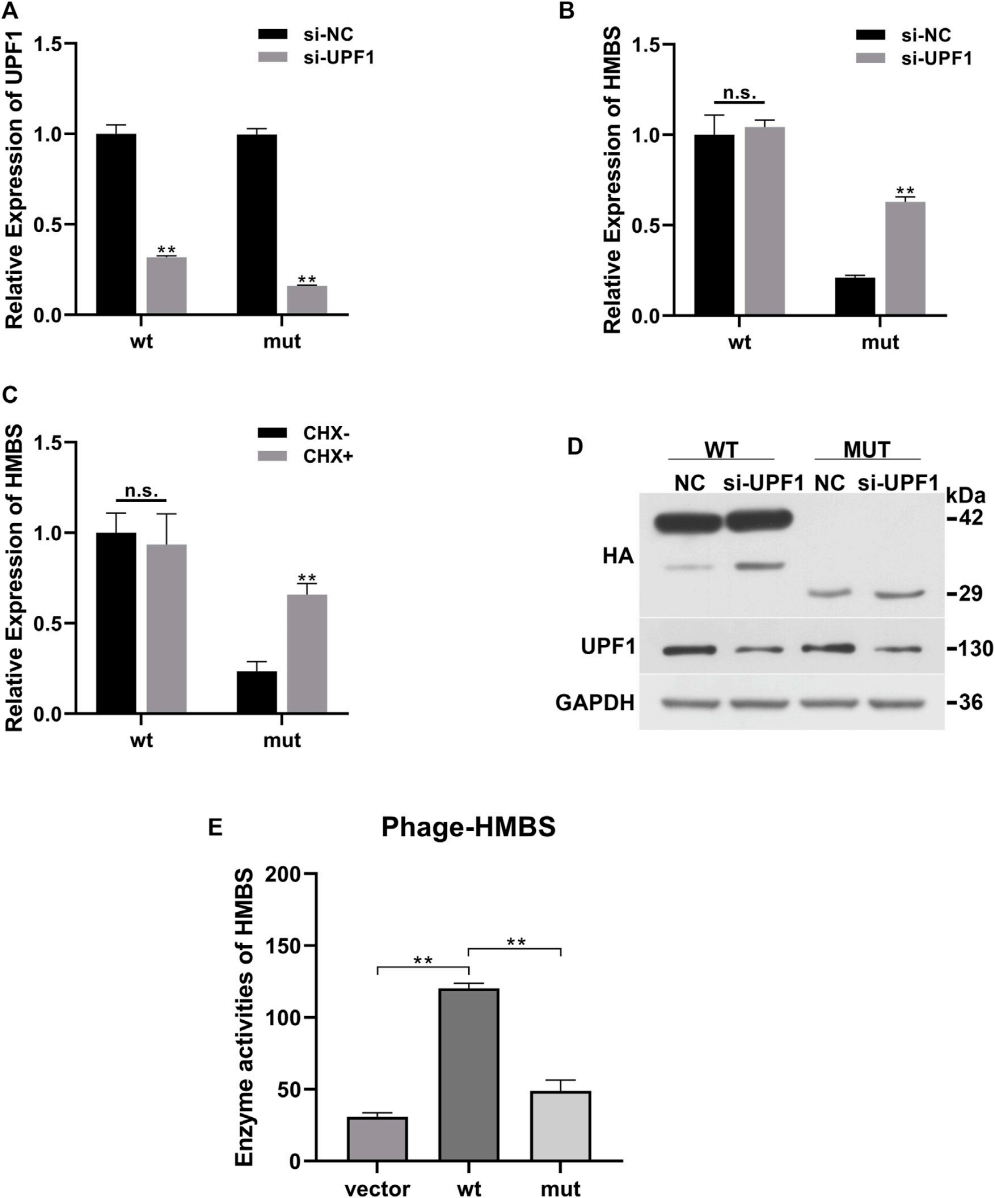
HMBS expression recovery results demonstration. **(A)** Compared with the siRNA negative control group, the relative expression of *UPF1* gene mRNA (QPCR) in the wild-type and mutant groups after interfering with *UPF1* gene; **(B)** The relative expression of *HMBS* gene mRNA (QPCR) in the wild-type and mutant groups before and after *UPF1* gene inhibition; **(C)** The relative expression of *HMBS* gene mRNA (QPCR) in the wild-type and mutant groups before and after CHX treatment; **(D)** The expression level of HMBS protein (WB) in the wild-type and mutant groups before and after *UPF1* gene inhibition; **(E)** HMBS enzyme activity assay.

Compared with the siRNA negative control (si-NC) group, the expression of UPF1 in the wt and mut cells was significantly downregulated (Figure 2A). Compared with the wt (si-NC) group, the mRNA expression of HMBS in the mut (si-NC) group was significantly downregulated; siRNA UPF1 treatment had no significant effect on HMBS mRNA expression in the wt group [wt (si-NC) vs. wt (si-UPF1)], but could significantly reverse the downregulation of HMBS mRNA expression in the mut group [mut (si-NC) vs. mut (si-UPF1)] (Figure 2B). Compared with the wt (CHX-) group, the mRNA expression of HMBS in the mut (CHX-) group was significantly downregulated; cycloheximide treatment (CHX+) had no significant effect on HMBS mRNA expression in the wt group [wt (CHX-) vs. wt (CHX+)], but could significantly reverse the downregulation of HMBS mRNA expression in the mut group [mut (CHX-) vs. mut (CHX+)] (Figure 2C).

UPF1 antibody was used to detect the expression level of UPF1 in the siRNA *UPF1* gene interference group (si-UPF1). The results showed that UPF1 was significantly inhibited (Figure 2D). The theoretical size of the HMBS protein in the wt group was 42 kd. After gene mutation, a truncated protein was produced, and the theoretical size of the mut protein should be 29 kd. The WB results showed that the sizes of the protein products were as expected. The expression level of HMBS in the mut group was significantly lower than that in the wt group [wt (UPF1-) vs. mut (UPF1+)]. Inhibition of the key component of the NMD pathway (UPF1) had no significant effect on the expression of HMBS in the wt group [wt (UPF1-) vs. wt (UPF1+)], and had significant effect on the expression of HMBS protein in the mut group [mut (UPF1-) vs. mut (UPF1+)] (Figure 2D).

The mRNA expression of wild-type *HMBS* was unaffected by knockdown of UPF1, a key component of the NMD pathway, but the degradation of mutated HMBS mRNA was significantly rescued, indicating that the HMBS mutant triggered the NMD. The results of two recovery experiments using CHX or UPF1 inhibition confirmed that the mutation-induced decrease in *HMBS* mRNA expression was due to the NMD degradation pathway. The mutation altered the HMBS protein structure. To test whether the mutant protein had residual enzyme activity, enzyme activity was detected by the human hydroxymethylbilane synthase enzyme activity detection kit. The enzyme activity results showed that the wt group had significantly higher enzyme activity than the Vector empty carrier group, while the mut group had significantly lower enzyme activity than the wt group, suggesting that the mutant protein lost almost all enzyme activity (Figure 2E).

### Structural analysis of HMBS protein

**FIGURE 3:**
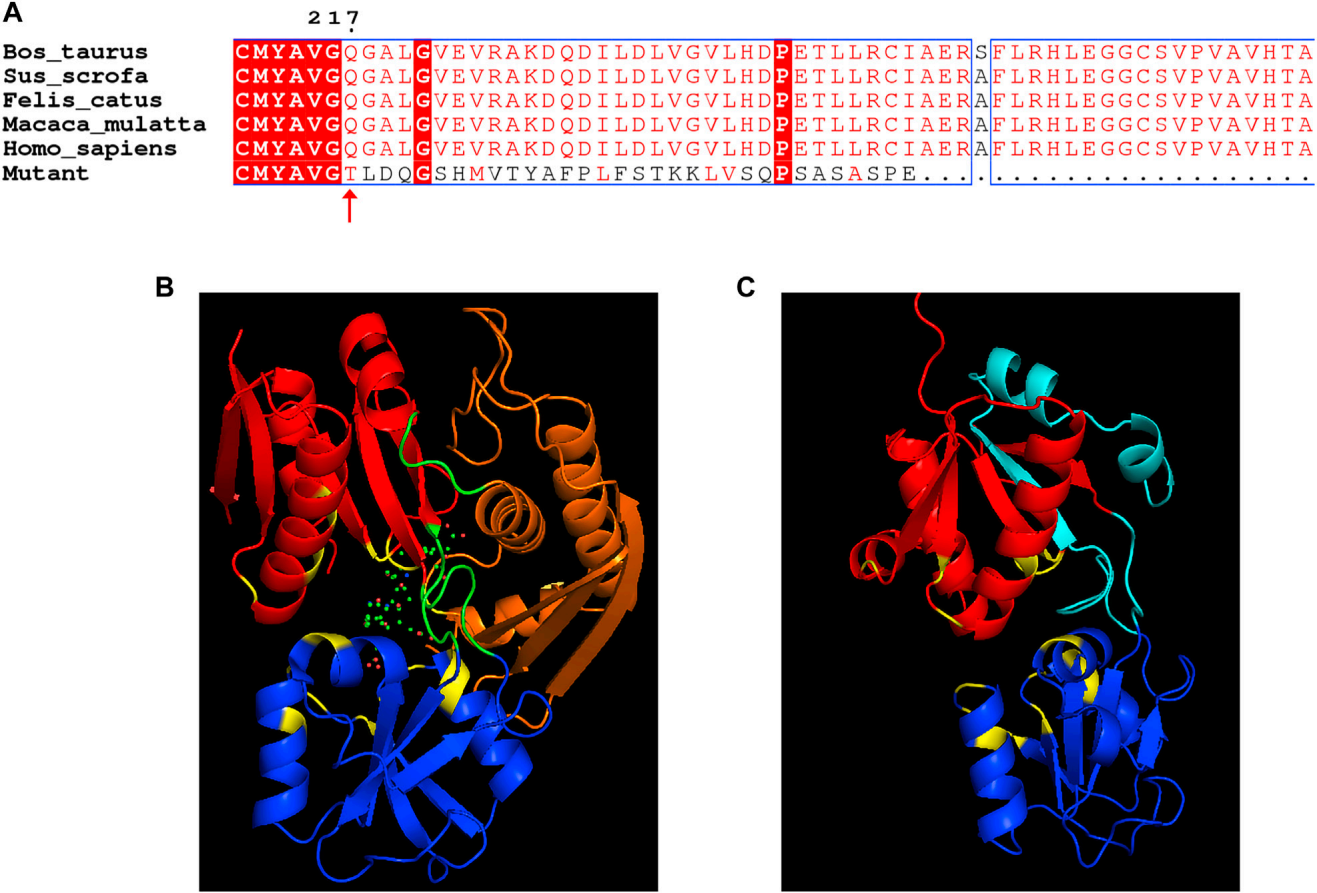
Analysis of *HMBS* mutations. **(A)** Evolutionary conservation of amino acid residues altered by c.648_651+1delCCAGG (p.Gln217Thrfs*33) across different species. NCBI accession numbers are *Bos taurus*: NP_001039672.1; *Sus scrofa*:NP_001090881.1; *Felis catus*: NP_001171279.1; *Macaca mulatta*: NP_001253589.1; *Homo sapiens*: NP_000181.2. (.) means different or missed amino acids. Ribbon representation of the HMBS-WT **(B)** and map of HMBS p.Gln217Thrfs*33 **(C)** by homology modeling analysis. HMBS is a monomer enzyme with three domains as pointed out. Domain 1 is shown in red, domain 2 is shown in blue and domain 3 is shown in orange. The critical active site is shown in yellow. K98, D99, T145, S147, R149, R150, R173, R195 were altered cofactor binding site; R26, S28, Q34, T58, S96, R167 were altered PBG binding site; S165, N169, S262 were affecting polypyrrole charge stabilization; R167 was product release blocked.

**FIGURE 4:**
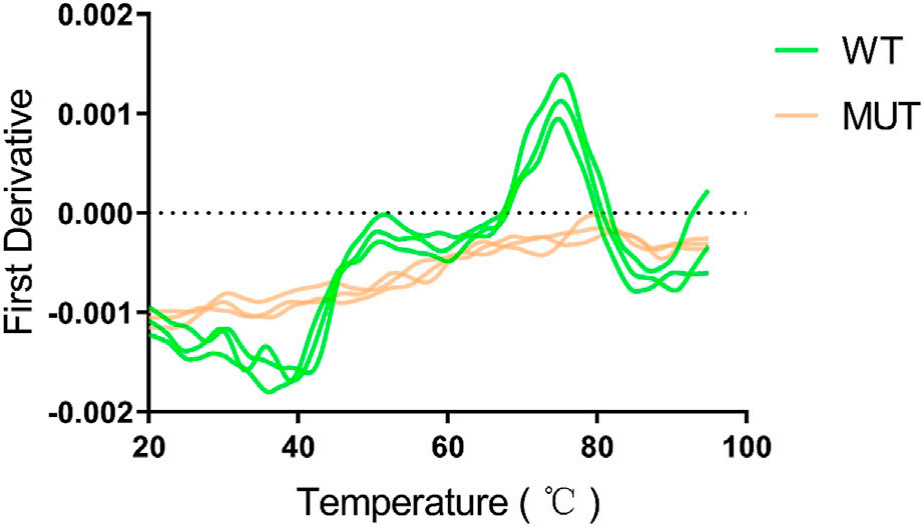
The p.Gln217Thrfs*33 mutant protein is heat intolerant. A obvious peak can be seen for the wild type protein, and the abscissa corresponding to the peak is the Tm value of 75°C ± 0.2°C. However, the mutant protein cannot be measured.

Human HMBS (hHMBS) has three different structural domains: domain 1 (residues 1 to 114, 219–236), domain 2 (residues 120–212) and domain 3 (residues 241–361), connected by interdomain hinge regions. HMBS enzymes have different structural and active site residues at different stages. At the dipyrromethene (DPM) cofactor stage, several polar and charged residues such as D99 form hydrogen bonds or ionic bonds with DPM cofactors to stabilize the structure. At the tripyrrole stage, several residues such as R26 interact with the carboxyl group of the newly added pyrrole ring C. At the tetrapyrrole stage, several residues such as T58 interact with the carboxyl group of pyrrole ring D. At the pentapyrrole stage, several residues such as S69 interact with the carboxyl group of pyrrole ring E. At the hexapyrrole stage, several residues such as S262 interact with the carboxyl group of pyrrole ring F (Bung et al., 2018). In addition, some water molecules stabilize the negatively charged polypyrrole chain through hydration. R26 and R167 are key residues for proton transfer and are involved in the deamination reaction of PBG. R167 is also the gatekeeping residue for the release of HMB from the enzyme. Mutations of these residues can affect the catalytic activity of the enzyme and lead to AIP. Through amino acid sequence alignment, we found that the c.648_651+1delCCAGG mutation resulted in the deletion of all amino acids after position 248 of the HMBS protein. The evolutionary conservation analysis of amino acid residues showed that the damaged amino acids were highly conserved during evolution in different species (Figure 3A). At the same time, protein structure analysis showed that, on the one hand, the defective HMBS protein lost HMBS domain 3 and was easily degraded. On the other hand, it lacked C261, which is critical for enzyme activity (Figure 3B). Thermal stability experiments also confirmed that the mutant protein was very unstable and degraded rapidly (Figure 4). Thermal stability assay showed that the Tm value of the p.Gln217Thrfs*33 mutant protein was almost undetectable, while the Tm value of the wild-type protein was 75°C ± 0.2°C.

## Literature review

Using ‘*HMBS*’ and ‘Acute intermittent porphyria’ as keywords, 19 English literature on clinical cases of AIP were retrieved from the PubMed database from 2013 to 2023 (Supplementary Table S1). These 19 literature reported a total of 220 HMBS gene mutations, of which 23 were found in China, 86 in the United States, 88 in Russia, and 23 in other countries. Some mutations were present in multiple countries. All mutation types included: missense mutations 68, splice site mutations 37, nonsense mutations 19, intronic mutations 9, deletion mutations 61, insertion mutations 26. Among the 19 literature, only 9 described the clinical manifestations of 10 patients in detail (Li et al., 2015; Balwani et al., 2016; Indika et al., 2018; Ren et al., 2018; Aksoy et al., 2020; Yang et al., 2020a; Yang et al., 2020b; Sriprakoon et al., 2022; Yusuf et al., 2023). The onset age of these 10 patients ranged from 9 to 32 years old. All patients had severe abdominal pain, nausea, vomiting and mental depression at the initial stage of the disease, and were often misdiagnosed as ‘intestinal obstruction’ or ‘gastroenteritis’. After hospitalization, the symptoms did not improve, and they developed recurrent abdominal pain, vomiting, seizures, hyponatremia and syndrome of inappropriate antidiuretic hormone secretion. They were diagnosed as acute intermittent porphyria after evaluation and their condition was controlled after targeted treatment. Other clinical symptoms included reversible posterior leukoencephalopathy syndrome, hypertension, anemia, vision loss, language and memory impairment.

## Discussion

Acute intermittent porphyria (OMIM#176000) is an inherited disorder caused by deficiency of hydroxymethylbilane synthase (HMBS; EC 2.5.1.61), the third enzyme in the heme biosynthetic pathway. During the characteristic acute attacks of this disease, toxic porphobilinogen (PBG) and 5-aminolevulinic acid (ALA) accumulate in tissues and cause damage to nerves and internal organs (Goncharova et al., 2019). So far, 517 *HMBS* variants have been reported in HGMD (version 2020.v2). Using Sanger sequencing, a new pathogenic splicing variant (c.648_651+1delCCAGG) was identified in this family. This mutation occurs in the junction region of exon 10 and intron 10. *In vitro* minigene expression also confirmed that this variant may lead to retention of intron 10 and aberrant splicing of mRNA (Zhou et al., 2022).

The *HMBS* gene consists of 14 exons and encodes 361 amino acids. A review of the locations of pathogenic variants shows that exon 12 is the exon with the most mutations, followed by exon 10 (Hu et al., 2022). The mutation c.648_651+1delCCAGG (NM_000190.4) was not found in the ExAC and gnomAD databases in the human population. The *HMBS* gene has a haploinsufficiency effect. This mutation is located in exon 10. Our results show that the mutant protein (29 kDa) was significantly lower than the wild type (45 kDa) and the expression level was significantly decreased, confirming that the variant caused a functional defect in the gene product. And the mutation caused NMD to occur. According to ACMG, it meets PVS1+PM2_Supporting and is classified as LP variant.

The biosynthesis of tetrapyrroles is essential for almost all forms of life on Earth. Tetrapyrroles can serve as precursors of heme, chlorophyll, cytochromes, and cobalamin (vitamin B12) and are involved in various biological processes (Hamza and Dailey, 2012). Human hydroxymethylbilane synthase is a monomeric enzyme that catalyzes the stepwise head-to-tail condensation of four porphobilinogen molecules to form the linear tetrapyrrole 5-hydroxymethylbilane (HMB). The human HMBS consists of three domains. Domain 1 is composed of two discontinuous regions comprising residues 1-116 and 216-239, domain 2 comprises residues 117-215, and domain 3 comprises residues 240-361 (Gill et al., 2009; Song et al., 2009). They are connected by flexible hinge regions (S96, H120, L238). Domain 3 interacts with structural domains 1 and 2 in the same way, playing an important role in maintaining the overall stability of the protein (Gill et al., 2009). Structural analysis of the aberrant HMBS protein (p.Gln217Thrfs*33) caused by the c.648_651+1delCCAGG mutation showed that the aberrant HMBS protein completely lacks domain 3, so it may be easily degraded and cause dose-insufficient effects (Gill et al., 2009). During catalysis, the active site region of hHMBS (i.e., the catalytic cleft) is located between domains 1 and 2. Arg26, Ser28, Ser96, Asp99, Lys98, Ser147, Arg149, Arg150, and Arg173 jointly form the catalytic active region where PBG binding and pyrrole elongation occur (Jordan and Warren, 1987; Louie et al., 1992). The DPM cofactor acts as a primer for HMB formation and is connected to the conserved C261 residue via a disulfide bond (Song et al., 2009). In addition, the extension of the pyrrole is catalyzed by HMBS using the same catalytic site in four single repetitive reactions. Reaction intermediates need to be transferred from the active site cleft in a timely manner, and the E250-C261 slide loop is closely related to this (Pluta et al., 2018). Therefore, E250–C261 is critical for reaction extension. The truncated protein produced by the mutant HMBS p.Gln217Thrfs*33 retains the first 217 amino acids of the original HMBS protein. Protein structural analysis results show that these deleted residues play an important role in the catalytic efficiency, substrate release, and protein stability of hHMBS. To confirm the inference, we measured HMBS protease activity. Compared with wild-type HMBS transfected cells, HMBS mutant transfected cells almost completely lost HMBS enzyme activity.

In summary, this study first demonstrated through *in vitro* experiments that the new splicing mutation (c.648_651+1delCCAGG) in the *HMBS* gene is pathogenic. Although describing a single pathogenic variant alone cannot fully explain the correlation between genotype and phenotype, the results of this study can provide new clues for understanding the pathogenesis of AIP and developing corresponding treatment methods.

## Conclusion

This study reported a 22-year-old female patient who developed an acute attack of AIP. By Sanger sequencing, we identified a novel splice site mutation (c.648_651+1delCCAGG) in the *HMBS* gene of the patient, which resulted in aberrant mRNA splicing and protein truncation. We verified the pathogenicity of this mutation by *in vitro* experiments, and found that it significantly reduced the expression and activity of HMBS, and degraded the mutant mRNA by NMD mechanism. Structural analysis predicted that this mutation would disrupt the stability and catalytic activity of HMBS protein. This study first identified and proved the pathogenicity of this novel splice site *HMBS* mutation (c.648_651+1delCCAGG), and elucidated the pathological mechanism by which it causes AIP through reducing HMBS expression and activity. These findings provide theoretical guidance for the diagnosis, treatment and genetic counseling of AIP patients.

## Data Availability

The original contributions presented in the study are included in the article/Supplementary Material, further inquiries can be directed to the corresponding authors.

## Abbreviations

AIP, Acute intermittent porphyria; ACMG, American College of Medical Genetics and Genomics; ALA, 5-aminolevulinic acid; CHX, Cycloheximide; DPM, Dipyrromethane; DSF, Differential scanning fluorimetry; ExAC, Exome Aggregation Consortium; gnomAD, Genome Aggregation Database; HGMD, Human Gene Mutation Database; HMB, 5-hydroxymethylbilane; HMBS, Hydroxymethylbilane synthase; NMD, Nonsense-mediated mRNA decay; OD, Optical density; PBG, Porphobilinogen; PBS, Phosphate-Buffered Saline; QPCR, Real-time Quantitative PCR; WB, Western blotting.

## References

[B1] Aksoy O. Y. Gunduz M. Unal O. Bostanci F. Cayci F. S. Bayrakci U. S. (2020). A mysterious case with abdominal pain and syndrome of inappropriate anti-diuretic hormone secretion. Turk J. Pediatr. 62, 487–490. 10.24953/turkjped.2020.03.018 32558425

[B2] Anderson K. E. Bloomer J. R. Bonkovsky H. L. Kushner J. P. Pierach C. A. Pimstone N. R. (2005). Recommendations for the diagnosis and treatment of the acute porphyrias. Ann. Intern Med. 142, 439–450. 10.7326/0003-4819-142-6-200503150-00010 15767622

[B3] Andersson C. Wikberg A. Stegmayr B. Lithner F. (2000). Renal symptomatology in patients with acute intermittent porphyria. A population-based study. J. Intern Med. 248, 319–325. 10.1046/j.1365-2796.2000.00743.x 11086643

[B4] Balwani M. Sardh E. Ventura P. Peiro P. A. Rees D. C. Stolzel U. (2020). Phase 3 trial of RNAi therapeutic givosiran for acute intermittent porphyria. N. Engl. J. Med. 382, 2289–2301. 10.1056/NEJMoa1913147 32521132

[B5] Balwani M. Singh P. Seth A. Debnath E. M. Naik H. Doheny D. (2016). Acute Intermittent Porphyria in children: a case report and review of the literature. Mol. Genet. Metab. 119, 295–299. 10.1016/j.ymgme.2016.10.005 27769855 PMC5154763

[B6] Baravelli C. M. Sandberg S. Aarsand A. K. Nilsen R. M. Tollanes M. C. (2017). Acute hepatic porphyria and cancer risk: a nationwide cohort study. J. Intern Med. 282, 229–240. 10.1111/joim.12646 28730628

[B7] Blaylock B. Epstein J. Stickler P. (2020). Real-world annualized healthcare utilization and expenditures among insured US patients with acute intermittent porphyria (AIP) treated with hemin. J. Med. Econ. 23, 537–545. 10.1080/13696998.2020.1724118 31999204

[B8] Bung N. Roy A. Chen B. Das D. Pradhan M. Yasuda M. (2018). Human hydroxymethylbilane synthase: molecular dynamics of the pyrrole chain elongation identifies step-specific residues that cause AIP. Proc. Natl. Acad. Sci. U. S. A. 115, E4071–E4080. 10.1073/pnas.1719267115 29632172 PMC5924904

[B9] Bustad H. J. Kallio J. P. Vorland M. Fiorentino V. Sandberg S. Schmitt C. (2021). Acute intermittent porphyria: an overview of therapy developments and future perspectives focusing on stabilisation of HMBS and proteostasis regulators. Int. J. Mol. Sci. 22, 675. 10.3390/ijms22020675 33445488 PMC7827610

[B10] Fontanellas A. Avila M. A. Anderson K. E. Deybach J. C. (2019). Current and innovative emerging therapies for porphyrias with hepatic involvement. J. Hepatol. 71, 422–433. 10.1016/j.jhep.2019.05.003 31102718

[B11] Fraile-Bethencourt E. Valenzuela-Palomo A. Diez-Gomez B. Caloca M. J. Gomez-Barrero S. Velasco E. A. (2019). Minigene splicing assays identify 12 spliceogenic variants of BRCA2 exons 14 and 15. Front. Genet. 10, 503. 10.3389/fgene.2019.00503 31191615 PMC6546720

[B12] Gill R. Kolstoe S. E. Mohammed F. Al D. B. A. Mosely J. E. Sarwar M. (2009). Structure of human porphobilinogen deaminase at 2.8 A: the molecular basis of acute intermittent porphyria. Biochem. J. 420, 17–25. 10.1042/BJ20082077 19207107

[B13] Goncharova M. Pshenichnikova O. Luchinina Y. Pustovoit Y. Karpova I. Surin V. (2019). Molecular genetic study of acute intermittent porphyria in Russia: HMBS gene mutation spectrum and problem of penetrance. Clin. Genet. 96, 91–97. 10.1111/cge.13558 31044425

[B14] Hamza I. Dailey H. A. (2012). One ring to rule them all: trafficking of heme and heme synthesis intermediates in the metazoans. Biochim. Biophys. Acta 1823, 1617–1632. 10.1016/j.bbamcr.2012.04.009 22575458 PMC3412874

[B15] Hu Y. Li W. Kang N. Ma L. Teng Q. Mo G. (2022). Identification and molecular analysis of 17 novel variants of hydroxymethylbilane synthase in Chinese patients with acute intermittent porphyria. Clin. Genet. 101, 116–121. 10.1111/cge.14063 34523126

[B16] Indika N. L. R. Kesavan T. Dilanthi H. W. Jayasena K. Chandrasiri N. Jayasinghe I. N. (2018). Many pitfalls in diagnosis of acute intermittent porphyria: a case report. BMC Res. Notes 11, 552. 10.1186/s13104-018-3615-z 30071891 PMC6071335

[B17] Jordan P. M. Warren M. J. (1987). Evidence for a dipyrromethane cofactor at the catalytic site of *E. coli* porphobilinogen deaminase. FEBS Lett. 225, 87–92. 10.1016/0014-5793(87)81136-5 3079571

[B18] Karim Z. Lyoumi S. Nicolas G. Deybach J. C. Gouya L. Puy H. (2015). Porphyrias: a 2015 update. Clin. Res. Hepatol. Gastroenterol. 39, 412–425. 10.1016/j.clinre.2015.05.009 26142871

[B19] Lenglet H. Schmitt C. Grange T. Manceau H. Karboul N. Bouchet-Crivat F. (2018). From a dominant to an oligogenic model of inheritance with environmental modifiers in acute intermittent porphyria. Hum. Mol. Genet. 27, 1164–1173. 10.1093/hmg/ddy030 29360981

[B20] Li Y. Qu H. Wang H. Deng H. Liu Z. (2015). Novel A219P mutation of hydroxymethylbilane synthase identified in a Chinese woman with acute intermittent porphyria and syndrome of inappropriate antidiuretic hormone. Ann. Hum. Genet. 79, 310–312. 10.1111/ahg.12107 25787008

[B21] Louie G. V. Brownlie P. D. Lambert R. Cooper J. B. Blundell T. L. Wood S. P. (1992). Structure of porphobilinogen deaminase reveals a flexible multidomain polymerase with a single catalytic site. Nature 359, 33–39. 10.1038/359033a0 1522882

[B22] Ma L. Tian Y. Qi X. Li P. Li J. Teng Q. (2022). Acute intermittent porphyria: prevalence of pathogenic HMBS variants in China, and epidemiological survey in Hebei Province, China. Ann. Transl. Med. 10, 560. 10.21037/atm-22-1600 35722412 PMC9201126

[B23] Pallet N. Mami I. Schmitt C. Karim Z. Francois A. Rabant M. (2015). High prevalence of and potential mechanisms for chronic kidney disease in patients with acute intermittent porphyria. Kidney Int. 88, 386–395. 10.1038/ki.2015.97 25830761

[B24] Park Y. Park J. Hwang H. J. Kim B. Jeong K. Chang J. (2020). Nonsense-mediated mRNA decay factor UPF1 promotes aggresome formation. Nat. Commun. 11, 3106. 10.1038/s41467-020-16939-6 32561765 PMC7305299

[B25] Pluta P. Roversi P. Bernardo-Seisdedos G. Rojas A. L. Cooper J. B. Gu S. (2018). Structural basis of pyrrole polymerization in human porphobilinogen deaminase. Biochim. Biophys. Acta Gen. Subj. 1862, 1948–1955. 10.1016/j.bbagen.2018.06.013 29908816 PMC6192514

[B26] Putscher E. Hecker M. Fitzner B. Lorenz P. Zettl U. K. (2021). Principles and practical considerations for the analysis of disease-associated alternative splicing events using the gateway cloning-based minigene vectors pDESTsplice and pSpliceExpress. Int. J. Mol. Sci. 22, 5154. 10.3390/ijms22105154 34068052 PMC8152502

[B27] Ramanujam V. S. Anderson K. E. (2015). Porphyria diagnostics-Part 1: a brief overview of the porphyrias. Curr. Protoc. Hum. Genet. 86, 17 20 1–17. 10.1002/0471142905.hg1720s86 PMC4640448 26132003

[B28] Ren Y. Xu L. X. Liu Y. F. Xiang C. Y. Gao F. Wang Y. (2018). A novel 55-basepair deletion of hydroxymethylbilane synthase gene found in a Chinese patient with acute intermittent porphyria and her family: a case report. Med. Baltim. 97, e12295. 10.1097/MD.0000000000012295 PMC6156069 30212967

[B29] Schmitt C. Lenglet H. Yu A. Delaby C. Benecke A. Lefebvre T. (2018). Recurrent attacks of acute hepatic porphyria: major role of the chronic inflammatory response in the liver. J. Intern Med. 284, 78–91. 10.1111/joim.12750 29498764

[B30] Schneider-Poetsch T. Ju J. Eyler D. E. Dang Y. Bhat S. Merrick W. C. (2010). Inhibition of eukaryotic translation elongation by cycloheximide and lactimidomycin. Nat. Chem. Biol. 6, 209–217. 10.1038/nchembio.304 20118940 PMC2831214

[B31] Song G. Li Y. Cheng C. Zhao Y. Gao A. Zhang R. (2009). Structural insight into acute intermittent porphyria. FASEB J. 23, 396–404. 10.1096/fj.08-115469 18936296

[B32] Sriprakoon V. Ittagornpunth C. Puapaiboon N. Bunyahathaipat A. Piriyanon P. Khositseth S. (2022). Acute intermittent porphyria: complete phenotype in a patient with p.Arg173Trp variant in Thailand. Am. J. Case Rep. 23, e937695. 10.12659/ajcr.937695 36329616 PMC9641550

[B33] Stolzel U. Doss M. O. Schuppan D. (2019). Clinical guide and update on porphyrias. Gastroenterology 157, 365–381. 10.1053/j.gastro.2019.04.050 31085196

[B34] Wang B. Rudnick S. Cengia B. Bonkovsky H. L. (2019). Acute hepatic porphyrias: review and recent progress. Hepatol. Commun. 3, 193–206. 10.1002/hep4.1297 30766957 PMC6357830

[B35] Whatley S. D. Badminton M. N. (1993). “Acute intermittent porphyria,” in GeneReviews((R)). Editors Adam M. P. Feldman J. Mirzaa G. M. Pagon R. A. Wallace S. E. Bean L. J. H. (Seattle (WA): National Library of Medicine).

[B36] Yang J. Han F. Chen Q. Zhu T. Zhao Y. Yu X. (2020b). Reversible splenial lesion syndrome (RESLES) due to acute intermittent porphyria with a novel mutation in the hydroxymethylbilane synthase gene. Orphanet J. Rare Dis. 15, 98. 10.1186/s13023-020-01375-y 32306994 PMC7168860

[B37] Yang Y. Chen X. Wu H. Peng H. Sun W. He B. (2020a). A novel heterozygous mutation in the HMBS gene in a patient with acute intermittent porphyria and posterior reversible encephalopathy syndrome. Mol. Med. Rep. 22, 516–524. 10.3892/mmr.2020.11117 32377710 PMC7248523

[B38] Yusuf A. Alhaj O. Aldaheri A. AlShamsi A. AlMarshoodi M. AlKindi F. (2023). Rare coexistence of acute intermittent porphyria with systemic lupus erythematous: case report and literature review. J. Investig. Med. High. Impact Case Rep. 11, 23247096231181856. 10.1177/23247096231181856 PMC10288411 37341437

[B39] Zhou Y. Q. Wang X. Q. Jiang J. Huang S. L. Dai Z. J. Kong Q. Q. (2022). Novel hydroxymethylbilane synthase gene mutation identified and confirmed in a woman with acute intermittent porphyria: a case report. World J. Clin. Cases 10, 12319–12327. 10.12998/wjcc.v10.i33.12319 36483813 PMC9724524

